# Perceived urban green and residents’ health in Beijing

**DOI:** 10.1016/j.ssmph.2021.100790

**Published:** 2021-04-08

**Authors:** Jingxue Xu, Fahui Wang, Li Chen, Wenzhong Zhang

**Affiliations:** a Key Laboratory of Region Sustainable Development Modeling, CAS, Beijing, 100101, China; b Institute of Geographic Science and Natural Resources Research, CAS, Beijing 100101, China; c University of Chinese Academy of Sciences, Beijing, 100049, China; d Department of Geography & Anthropology, Louisiana State University, Baton Rouge, LA, 70803, USA; e College of Arts and Sciences, Beijing Union University, Beijing, 100191, China

**Keywords:** Perceived urban green, Park green, Public-square green, Utility green, Health, Beijing

## Abstract

Green space serves urban residents in various functions including promoting health, but the roles of different types of green space are unclear. A survey titled “Healthy Neighborhood” was conducted in Beijing from May to July 2019 to examine and compare the associations between three types of perceived green space (park green, public-square green and utility green) and three aspects of residents' health (physical health, mental health and social health). Results from the multilevel modeling (MLM) analysis show that the perceived park green has a positive correlation with mental health, and all three types of perceived green space correlate with social health positively. No significant correlation of any type of green space is detected on participants’ physical health, nor any relation of public-square green or utility green to their mental health. Overall the role of urban green space is stronger on social health than physical and mental health. The findings support the complementary roles of different types of green space, and suggest that expansion in utility greens could be as effective as investing in more costly park and public-square greens, especially in their benefit in promoting social health.

## Highlights

• Green space serves residents in various forms, and different types of green space affect residents' health differently.
• The role of urban green space is stronger on social health than physical and mental health.
• Expansion in utility greens could be as effective as investing in more costly park and public-square greens.

## 1 Introduction

China's rapid economic growth and massive urban development in the past four decades or so have come with major changes to the urban lifestyle that is now fast paced, with high pressure, and increasingly detached from nature. The lifestyle leads to rising fatigue, stress, depression, anxiety and other unhealthy indicators among city dwellers (Gong et al. 2012), and many suffer from chronic diseases, psychological disorders, and social adaptation challenges. In response, the central government of China launched the “Healthy China 2030” initiative in October 2016 (Tan et al. 2017). Since then, many cities have followed up with plans toward the goal of a healthy city, and Beijing has been leading the charge (Yang et al. 2018). One major strategy of healthy city movement focuses on the preservation and expansion of urban green space.

Urban green space is critical to a healthy city (Wolch et al. 2014), and offers much needed counter balance to the negative effects of rapid and unsustainable urbanization on residents’ health and well-being (Röbbel, 2016). However, urban greening in China has its unique challenges, and foremost, green space is usually insufficient, highly unbalanced and uncoordinated in cities in China (He et al. 2020). In 2018, the green coverage rate of urban built-up areas was only 41.1%, and the public recreational green space per capita was just 14.11 square meters in China (NBSC 2019). China remains far behind developed countries, with severe deficiency of supply in green space (Russo et al. 2018).

According to the WHO (2016), environmental conditions are a significant determinant of population health, and vary across geographic areas and population groups. Socioeconomically disadvantaged groups tend to be disproportionally concentrated in areas with poor environmental conditions. In urban areas, access to green spaces has increasingly become an environmental justice issue. Increasing inequality in exposure to green spaces exacerbate health risks to these disadvantaged groups (Hoffimann et al. 2017), even more so in densely inhabited inner cities in China (Sun et al. 2019).

To mitigate this major public health and environmental justice problem, urban planners and policy makers need to be conscious of different types of green space and their corresponding functions including health benefits. How can we make the best use of available space, and what type of green space do we preserve or convert to? Answers to these questions rely on a solid understanding of the residents' perception of green space and related health impacts. In the meantime, the types of green space need to be defined and their distinctive roles need to be clarified. Much of the existing literature on the relationship between urban green space and residents' health has focused on theoretical and methodological issues (Markevych et al. 2017), and often on a single type of green space such as parks (Lee & Maheswaran, 2011; Wang & Lan, 2019; Wood et al. 2017). Few studies examine the associations between different types of urban green space and different aspects of residents’ health.

In short, green space serves urban residents in various forms, and different types of green space have different associations with residents' health. The key pathway is how residents perceive and use each type of green space. It is critical to identify whether a type of green space is related to residents’ health and which type of green space has the strongest connection. Findings from the study will have significant implications for planning an urban green space system, particularly in a compact city such as Beijing, toward the overall goal of building healthy cities in China.

## 2 Literature review

Health is traditionally a physiological concept for the human body (Nordenfelt, 2018). With the ever expansion of economic growth and accompanied needs of social development and progress, our connotation of health continues to evolve. Since broadening the definition by the WHO in 1948, health has been viewed more broadly (Seymour, 2016). It is a state of complete physical, mental, and social well-being, beyond the absence of disease or infirmity (Huber et al. 2011). Therefore, health in the modern era includes three dimensions: physical health, mental health, and social health (WHO 2010). *Physical health* is defined as a capability that when confronted with a physiological stress, a healthy organism can mount a protective response to mitigate the potential for harm and restore the body to an equilibrium (Huber et al. 2011). *Mental health* is a state of well-being in which an individual can realizes their abilities, cope with the normal stresses of life and work productively (WHO 2010). *Social health* refers to one's ability of maintaining good interpersonal relationships and social adaptation (Zhang et al. 2018). One's physical and mental health may collectively affect their social health (Thoits, 2011), and so does social health influence physical and mental health (Tough et al., 2017). While the concept of social health has been widely used since its inception by WHO (2010), it specific measurement was a fairly recent endeavor (Zhang et al. 2018, 2019) by refining the Social Cohesion and Support Scale developed by sociologists (Sampson et al., 1997; Völker et al., 2007).

Green space can positively relate to all aforementioned dimensions of health through various pathways (Bowler et al. 2010; James et al. 2015; Zhang et al. 2020). First of all, urban green space plays a prominent role in maintaining biodiversity, improving urban micro climate, and absorbing pollutants (Heidt & Neef, 2008; Vargas-Hernández et al. 2017). In the context of climate change, with the expected increase in temperature, dryness and intensity of heat waves, green spaces assume even higher importance as they provide shading and evaporative cooling to reduce daytime urban surface temperatures (Arifwidodo & Chandrasiri, 2020; Connors et al. 2013; Oliveira et al. 2011). Our study area, Beijing, is no exception to the increasing prevalence of urban heat island (Yao et al. 2020). In winter when the heat island effect is most prominent, the temperature difference between urban and rural areas at night is as high as 8 °C (Cui et al. 2017). In summer when its adverse effects on residents’ health are most pronounced, urban greening can change the thermal properties of underlying surface and reduce the accumulation of heat, and thus plays a key role in reducing its negative effect. All these ecological benefits directly improve the physical health of residents (Kondo et al. 2018).

Green space also plays a positive role in improving residents’ lifestyle such as more physical activity, better mental health, and increased social interaction (Europe 2017). Green space provides safe, low-cost, and attractive places to exercise and promotes physical activity (Douglas et al. 2017; Wang, Dai, et al., 2019). Physical activity then improve their physical and mental health (Biddle, 2016; Lahart et al. 2019; Warburton et al. 2006), and helps residents recover from fatigue and reduce stress (Berto, 2014). Views of nature have been related to increased feelings of peace, escape from distraction, and neighborhood satisfaction. Moreover, green space in a neighborhood is one of the few congregation places where urban residents can have direct and sustaining contact with nature (James et al., 2015), and facilitate social interactions and cohesion among residents (Jennings et al. 2016, 2019; Peters et al. 2010). If people are drawn to green space for health benefits, they are likely to meet other people seeking the same relaxation and restoration (Holtan et al. 2015), and due to the increased use of the green spaces, which then led to stronger social ties.

However, in order to cultivate the health benefits of green space, residents need to develop a positive perception of green space so that they can consciously engage with it (Bloemsma et al. 2018; Fongar et al. 2019). The perception influences a user's motivation, preferences and attitudes (Nasar, 2008). Those who find green spaces attractive, pleasant, and safe are more likely to use them. On the contrary, those who feel it unsafe or of low quality tend to avoid them (Jim & Shan, 2013; Russo & Cirella, 2018). Therefore, it is critical to assess the perception of green space by local residents (Ives et al. 2017).

Despite a growing body of literature on the relationship between health and green space, there is no consensus on how to measure exposure and access to green space properly (Xiao et al. 2019). One approach distinguishes objective vs. subjective measures of green space. Traditional objective measures include size, normalized difference vegetation index (NDVI), greening rate, proximity, and accessibility to quality green space (Akpinar et al. 2016; Ekkel et al. 2017; Nutsford et al. 2013; Reid et al. 2018; Zhang et al., 2020). Some of those measures are based on data of inadequate resolutions, use poorly-conceived accessibility measures, ignore a user's self-movement and perception, and lack a comprehensive picture of green space properties (Wendelboe-Nelson et al. 2019). Subjective measures focus on the perception of green space by local residents (Haslauer et al. 2015; Kothencz et al. 2017; Lee & Maheswaran, 2011; Sefcik et al. 2019). Such measures are often acquired by well-designed questionnaires to identify what elements of green space are valued or not valued by residents, and thus potentially form a more reliable and direct gauge on the pathway from green space to health.

A recent study by Zhang et al. (2019) used both objective and subjective measures to analyze the associations between neighborhood environment and residents across physical, mental and social health in Guangzhou, a southern city of China. Their study includes green space as a major component of neighborhood environment, but did not differentiate green space types. Not all green spaces are equal. As noted by Wolch et al. (2014, p.237), many areas of green space in Chinese cities are small and do not have facilities to promote “active recreation.” While the government has more control of land, urban greening in China shares similar market incentives with western cities. Greening can be very expansive in major cities in China, and land use planning including green space is subject to strict zoning restrictions. According to the official document CJJ/T 85–2017 released by the Ministry of Housing and Urban-Rural Development of China (MOHURD 2017), there are three types of urban green space: park green (G1), utility green (G2), and public-square green (G3) (authors’ translation). Each green space type is subject to its own guidelines, and no green space is cheap. Urban planner and policy makers need to ask what type is affordable, suitable and most valued by local residents, and how the perception of residents vary by their socioeconomic and demographic groups.

In short, it is important to understand that various types and sizes of green space function differently by design, and their associations with health may also differ. This paper is the first to investigate how each type of green space (park, utility, and public-square green) are related to which dimension of health (physical, mental and social health) differently. The study is based on a survey conducted in the summer of 2019 in Beijing. We aim to help advance the strategies of developing urban green space beyond what are ‘just green enough’ (Curran & Hamilton, 2012), toward “what type of green.”

## 3 Study area, data and variable definitions

### 3.1 The Healthy Neighborhood Survey

**Fig. 1:**
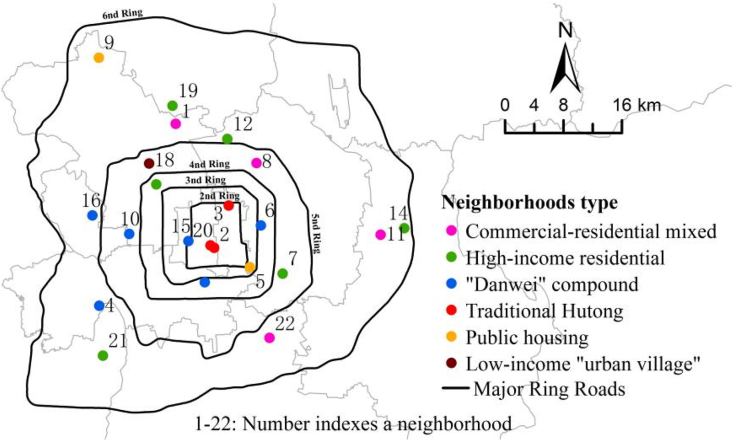
Locations of 22 sampled neighborhoods in Beijing.

The study area is Beijing, the capital city of China. By the end of 2019, Beijing had a population of approximately 21.54 million, with a density of 1313 persons per square kilometer (BMBS 2019). Data for this study is based on the Healthy Neighborhood Survey conducted in Beijing from May to July of 2019 by the research team. The questionnaire survey was approved and sponsored by the Beijing Municipal Institute of City Planning and Design (BICP) and the Institute of Geographic Sciences and Natural Resources Research of Chinese Academy of Sciences (IGSNRR). The survey investigated the associations between green spaces and residents’ self-reported physical health, mental health, and social health in neighborhoods. The survey was designed to follow a random stratified sampling strategy. Specifically, 22 sample neighborhoods from 10 districts within the Sixth Ring Road (Fig. 1) were selected to represent a diverse set of neighborhoods, such as commercial-residential mixed land use area, high-income residential area, work-unit (“Danwei”) compound, traditional Hutong residential area, public housing area, and low-income “urban village” area (Table 1). The research team worked closely with the survey contractor, ePanel Inc. (epanel.cn/research.cn), to implement the survey. The participants were limited to adults (>21 years old) who had lived in the neighborhood for more than six months. 60 residents were recruited from each neighborhood, and a total of 1320 participants were interviewed by a team of trained survey managers. Each interviewee received a gift of bath towel for their participation. A total of 1152 valid questionnaires, representing a wide spectrum of sociodemographic groups (Table 3), were finally obtained with an effective returning rate of 87.27%.

**Table 1:** List of sampled neighborhoods.

Neighborhood type	Neighborhood index number and name
Commercial-residential mixed	1 Anningli, 8 Nanhuzhongyuan, 11 Tianqiaowan, 22 Zhongxingxincheng
High-income residential	7 Jinchannali, 12 Wankexingyuan, 13 Wanquanxinxinjiayuan, 14 Wuyihuayuan, 19 Xinlongcheng, 21 Changyangbandao
“Danwei” compound	4 Hechenggong, 6 Hujialoubei, 10 Sanjiefangxi, 15 Tiedaobu, 16 Xijing, 17 Xilidier,
Traditional Hutong	2 Dashilanxijie, 3 Guozijian, 20 Yanshoujie
Public housing	5 Hongshanjiayuan, 9 Sanjiaxinyuan
Low-income “urban village”	18 Guajiatun

### 3.2 Individual health outcome variables

Outcome variables included self-reported physical health, mental health and social health. All were measured as participants’ personal subjective feelings of each dimension of health. Questions for physical and mental health were from the Health Questionnaire of Urban Residents in China (Table 2), developed by the Center for Health Education of China. As stated in the literature review, recent studies (Zhang et al. 2018, 2019) have developed a specific metric to measure social health by capturing the sense of belonging to and trust in their neighborhood, supported by the literature for conceptualizing neighborhood cohesion (De Vries et al., 2013; Robinson & Wilkinson, 1995). Following the five questions proposed in Zhang et al., 2018, this study refines the list with five similar questions (Table 2). We designed the questions for measuring social health in the neighborhoods. Answer to each question was rated on a 1–5 Likert scale (1 for the least healthy and 5 for the healthiest). The total score (1) for physical health status with seven questions ranged from 7 to 35, (2) for mental health status with 13 questions ranged from 13 to 65, and (3) for social health with five questions ranged from 5 to 25. The mean scores for physical, mental and social health were 24.45, 46.12 and 17.97, respectively.

**Table 2:** Health outcome measures.

Outcome variables (number)	Questions	Mean score	Cronbach's α	Kaiser-Meyer-Olkin
Physical health (7)	Do you feel tired and weak? Do you have a headache, low back pain, or muscle pain? Do you feel dizzy? Do you experience excessive sweating (excluding seasonal or other external factors)? Do you feel palpitating and short of breath after light exercise? Do you feel any gastrointestinal discomfort? Do you have low immunity?	24.45	0.855	0.897
Mental health (13)	Do you find it difficult to concentrate? Do you feel memory loss? Do you feel unresponsive? When you are doing things, are you prone to hesitation and indecision? Are you unable to control your emotions and easy to lose your temper? Are you upset all the time? Do you feel no future or hope for you? Do you feel more nervous and anxious than before and cannot relax? Are you worried about things now or in the future? Do you feel that you do not want to do anything? Do you feel powerless when doing things? Have you lost sleep (insomnia or drowsiness)? Do you feel dizzy and lack of energy after getting up in the morning?	46.12	0.914	0.940
Social health (5)	Are you satisfied with the interaction with your neighbor? Are you satisfied with the manners of residents in your neighborhood? Are you satisfied with the property management of your subdivision? Are you satisfied with the community participation in your neighborhood? Are you satisfied with the community attachment?	17.97	0.743	0.784

**Table 3:** Basic statistics of individual socio-demographic variables.

Variables	Category (mean %)
Age	<30 (24.4%), 30–39 (21.5%), 40–49 (17.6%), 50–59 (15.9%), 60+ (20.6%)
Gender	Male (55.1%), Female (44.9%)
Education	No college degree (72.1%), College degree (28.0%)
Employment status	Employees in formal sectors (49.2%), Self-employed (3.4%), Freelancer (9.7%), Unemployed (4.9%), Retiree (29.4%), College student (3.4%)
Annual household income (RMB)	<100k (37.9%), 100k~199k (38.1%), 200k~299k (12.7%), 300k~499k (8.1%), 500k+ (3.2%)
Residence status	Beijing permanent resident (64.0%), Non-permanent resident (36.0%)
Housing tenure	Renter (34.8%), Homeowner (65.2%)

Based on both the Cronbach's α and the Kaiser-Meyer-Olkin test as reported in Table 2, the variables designed were reliable and captured distinctive traits of health status.

### 3.3 Individual socio-demographic variables

The explanatory variables at the individual level were mainly the demographic and socioeconomic characteristics of residents. The demographic variables included age, gender, marital status, and residence status (permanent resident^1^ of Beijing or else), and the socioeconomic characteristics are annual household income, education attainment, employment status, and housing tenure (renter or homeowner). Table 3 outlines the basic statistics of these variables.

### 3.4 Neighborhood green space perception by residents

As stated previously, urban green space is divided into three categories such as park green (G1), utility green (G2) and public-square green (G3), all closely related to residents’ daily life.^2^ Table 4 outlines the guideline for the classifications. For detailed classification codes and standards, refer to the Standard for Classification of Urban Green Space or CJJ/T 85–2017 by the Ministry of Housing and Urban-Rural Development (MHURD) of China (2017).

**Table 4:** Urban green space perception by residents.

Type	Standard for classification	Mean perception score
Park green (G1)	Refers to parks open to the public and with facilities for sight-seeing, recreation, entertainment, etc. Greening rate ≥65%.	3.74
Utility green (G2)	Refers to green belt and green land used as transition between land uses, sanitation, safety, and disaster mitigation, etc.	3.75
Public-square green (G3)	Refers to public event venues for recreation, commemoration, assembly & disaster mitigation. Greening rate ≥35%	3.85

Each participant was first asked the question: how satisfied are you with the park green in your neighborhood? The same question was repeated for public-square green, and then for utility green. The answer was given on a 5-point Likert scale, ranging from 1 (very dissatisfied) to 5 (very satisfied). If a respondent chose the answer “very dissatisfied” or “unsatisfied,” the interviewer would continue to ask the specific reasons for that answer. Individual ratings within a neighborhood were averaged as the overall satisfaction level at the neighborhood level.^3^ Table 4 reports the average of all respondents’ ratings for each green type.

## 4 Research design

**Fig. 2:**
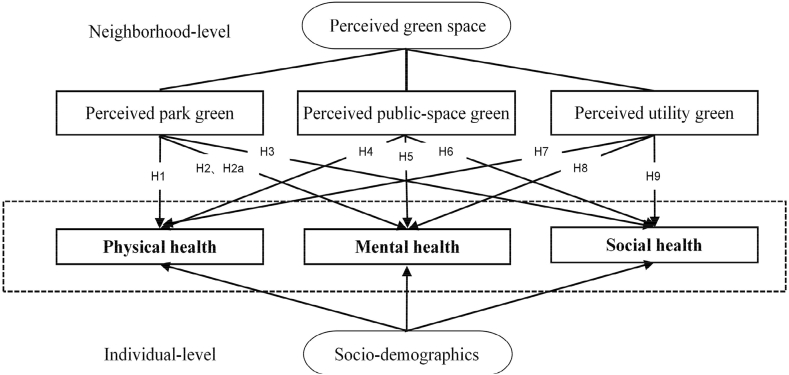
Conceptual framework and nine hypotheses.

As stated previously, green space may play an essential role in promoting the health of urban residents. This study defines three types of urban green space and examines the association between each type of green space and each health benefit. As shown in Fig. 2, the conceptual framework illustrates the joint relationships of neighborhood-level green space and individual attributes on individual health status (physical health, mental health, and social health). The study tests nine hypotheses on whether each of the three health statuses is related to each of the three green space types.

The multilevel modeling (MLM) is used to test these hypotheses. Individual health behavior or outcome is usually a result of combined effects from both individual and neighborhood factors (Wang, 2020). Since individuals are nested within their neighborhoods, it is appropriate to use the MLM for estimating the associations (Maas et al. 2006, 2008; Van Dillen et al. 2012; Astell-Burt and Feng 2019; Yang et al. 2019). It not only models and tests the associations between the individual-level and neighborhood-level variables, but also provides variance components of these two levels (Snijders & Bosker, 2011).

The following steps are used to implement the analysis:

1) Test the multi-collinearity of independent variables. A high correlation between them may suggest the need to build separate models for different neighborhood-level variables.
2) Build null models to test whether it is necessary to use the MLM. Specifically, when the intra-class correlation coefficient (ICC) at the neighborhood level is larger than 5.9%, the use of MLM is warranted (Cohen, 2013).
3) Use OLS regressions to examine the associations between only individual-level variables and individual health outcomes to establish a baseline.
4) Use the MLM to examine the relationships between the individual-level and neighborhood-level variables and individual health outcomes, that is, test the nine hypotheses as shown in Fig. 2.

## 5 Results

### 5.1 Association between self-rated health and perceived green space

**Fig. 3:**
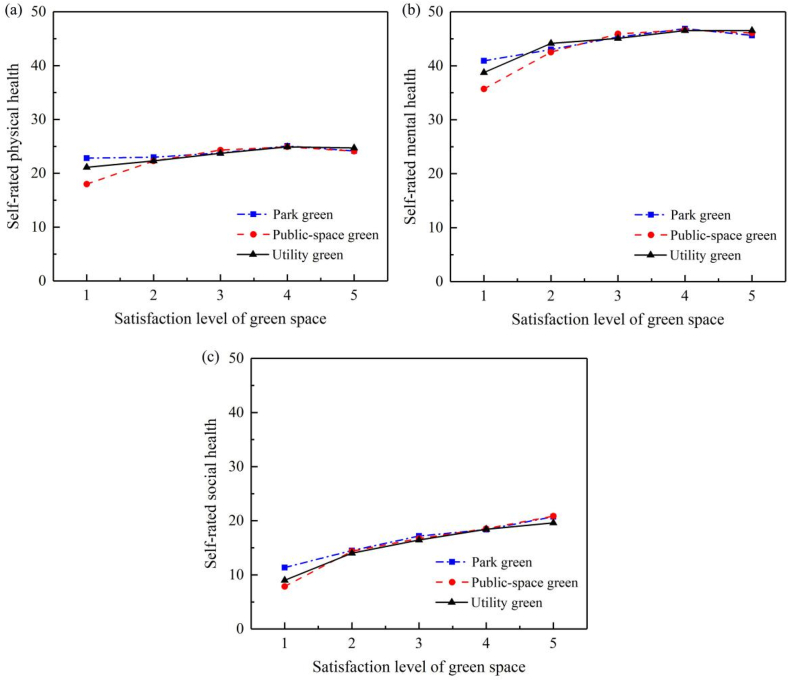
Residents' self-rated (a) physical health, (b) mental health, and (c) social health vs. perceived green space.

A single factor analysis of variance (one-way ANOVA) is used to test whether there are significant differences in self-rated health among groups with different types of perceived green space. Fig. 3 illustrates residents' self-rated health at different levels of exposure to perceived green spaces, with a 99 percent confidence interval. Note that as shown in Table 2, the mean scores for physical, mental and social health are 24.45, 46.12 and 17.97, respectively. The gaps between the highest and lowest scores in social health are the largest across three types of perceived green. The scores of the three dimensions of health tend to increase with the increase of residents’ satisfaction level with three types of green space. There are some exceptions between the perceived green space level 4 (satisfied) and 5 (very satisfied), where the order of corresponding self-rated health levels is reversed. However, the overall trend is largely consistent.

**Fig. 4:**
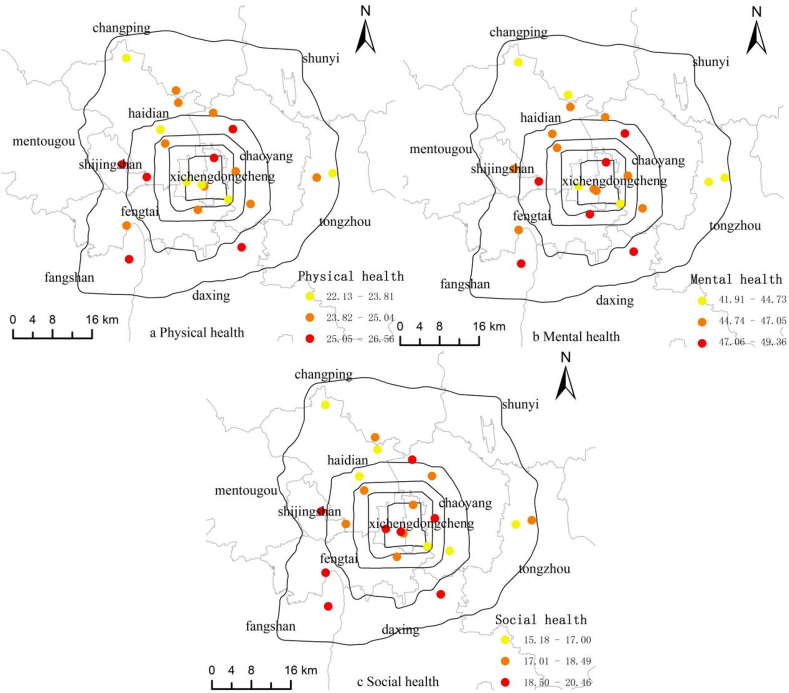
Self-rated (a) physical health, (b) mental health, and (c) social health in Beijing.

Fig. 4 shows the spatial variations in physical health, mental health, and social health across the 22 sampled neighborhoods. Both the highest levels of physical and mental health are found in Zhongxingxincheng (26.56 and 49.36), a commercial-residential mixed neighborhood in Daxing District. Both the lowest physical and mental health scores are in Sanjiaxinyuan (22.13 and 41.91), a public housing neighborhood in Haidian District. The highest level of social health is in Yanshoujie (20.46), a traditional “hutong” residential neighborhood in Xicheng District, and the lowest level is again in Sanjiaxinyuan (15.18). One may speculate whether participants with worse self-rated health in all three dimensions are more likely to live in neighborhoods of public housing (or low-income “urban village”), and whether neighborhoods of traditional hutong or “Danwei” compound help facilitate social interaction and promote better social health. The differentiation of neighborhood types is a manifestation of the differentiation of urban social space, and may exacerbate health inequality. This waits to be validated by more rigorous analysis in future work.

**Fig. 5:**
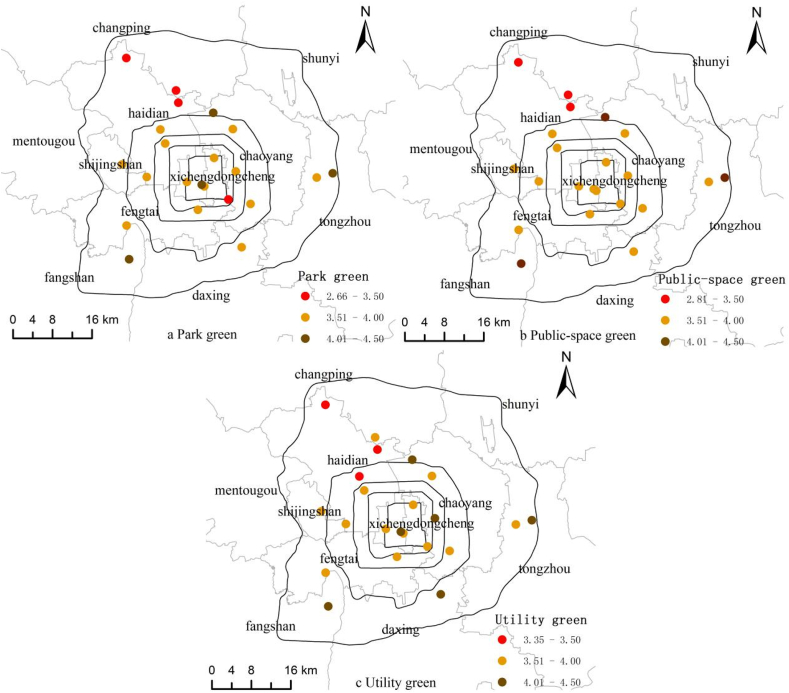
Perceived levels of (a) park green, (b) public-square green, and (c) utility green in Beijing.

Fig. 5 shows the perceived levels of park green, public-square green and utility green among the 22 sampled neighborhoods. The satisfaction levels of these three green spaces across neighborhoods are highly correlated. For example, the Wuyihuayuan, Wankexingyuan, and Changyangbandao Neighborhoods receive rates higher than 4 for all three green types, while the Sanjiaxinyuan Neighborhood scores at the bottom in all three green types. Other neighborhoods receive low ratings include: park green <3.5 in Xinlongcheng, Hongshanjiayuan and Anningli, public-square green <3.5 in Xinlongcheng and Anningli, and utility green <3.5 in Anningli and Guajiatun.

Table 5 lists the aforementioned five neighborhoods with low perceived levels of green space. Examining the leading causes of dissatisfaction among survey respondents yields two popular reasons: small size of and long distance from the green space.

**Table 5:** Frequency for the leading causes of dissatisfaction among survey respondents.^4^

Neighborhood	Park Green				Public-square Green			
	SS	LD	PQ	HC	SS	LD	PQ	HC
Anningli	12	10	1	0	9	4	0	0
Hongshanjiayuan	2	1	0	0	2	0	0	0
Sanjiaxinyuan	18	8	0	1	14	5	0	1
Guajiatun	3	0	0	0	5	1	0	0
Xinlongcheng	8	2	0	0	7	0	0	0

Note: SS for small size, LD for long distance, PQ for poor quality, and HC for high cost.

In general, the statuses of physical, mental, and social health of residents vary significantly across the neighborhoods, so do the perceived levels of three types of green space. Overall, better self-rated health statuses tend to be related to higher ratings of green space. Rigorous statistical analysis is needed to verify whether such a relationship is consistent across different types of health and different categories of green space, and whether the association remains after controlling for the effects of individual variables.

### 5.2 Variance component analysis in the null models

The null models are constructed without any individual-level variables to examine the extent to which variance in the outcome variables can be explained by the differences in neighborhood-level variables. The results in Table 6 show that the differences between neighborhoods can explain 6.1%,7.2%, and 12.8% of the differences in residents’ physical health, mental health, and social health, respectively. Since all the intraclass correlation coefficients (ICC) are higher than 0.059, the differences in physical health, mental health, or social health are all impacted by a combination of individual socioeconomic attributes and neighborhood environments, and thus warrant the use of MLM.

**Table 6:** Variance component analysis in the null models.

Outcome variables	Level	Variance Component	Intraclass correlation coefficient	Chi-square
Physical health	Individual-level	14.023	93.9%	
	Neighborhood-level	0.911	6.1%	81.507***
Mental health	Individual-level	45.775	92.8%	
	Neighborhood-level	3.569	7.2%	109.210***
Social health	Individual-level	9.785	87.2%	
	Neighborhood-level	1.441	12.8%	178.215***

*** *P* < 0.001.

### 5.3 Associations between perceived green space and health

Table 7 shows the analysis results with only individual-level socio-demographic variables as explanatory variables. Table 8 shows the MLM analysis results of testing hypotheses H1–H9 (as outlined in the conceptual framework in Fig. 2). As the effects of the individual-level variables are fairly consistent between the models for corresponding outcome variables in Table 7, Table 8, the analysis results for the individual-level variables are omitted in Table 8.

**Table 7:** Modeling associations between individual-level variables and health by OLS regression.

Outcome variable	Physical health	Mental health	Social health
Intercept	24.778*** (0.487)	46.132*** (0.440)	18.026*** (0.266)
Individual-level variables			
Gender (reference group:Female)			
Male	1.075** (0.303)	2.122*** (0.498)	−0.537** (0.216)
Age (reference group: <30)			
30–39	−0.392 (0.379)	−0.425 (0.766)	−0.052 (0.301)
40–49	−0.912** (0.402)	−2.232** (0.804)	−0.194 (0.423)
50–59	−0.979** (0.443)	−2.158** (0.866)	−0.491 (0.597)
60+	−1.045** (0.179)	−1.998* (1.048)	0.200 (0.599)
Income (RMB) (reference group:<100k)			
100k-199k	0.879** (0.423)	1.745** (0.574)	−0.101 (0.235)
200k-299k	0.282 (0.534)	0.765 (0.836)	0.433 (0.275)
300k+	0.806 (0.537)	1.953** (0.805)	0.215 (0.252)
Education (reference group:No college degree)			
College degree	0.186 (0.292)	−0.227 (0.447)	−0.192 (0.222)
Employment status (reference group:formal-sector employees)			
Self-employed	1.603** (0.393)	2.166*** (0.625)	−1.071** (0.523)
Freelancer	−0.049 (0.502)	−0.560 (0.675)	−1.039* (0.561)
Retiree	−0.888 (0.543)	−1.307 (1.031)	−0.793 (0.528)
Unemployed	−0.632 (0.924)	−0.921 (1.549)	−1.311** (0.446)
College student	0.854** (0.413)	1.917** (0.883)	0.895 (0.720)
Marital status (reference group: Unmarried)			
Married	0.411 (0.314)	0.778 (0.519)	0.249 (0.328)
Residence status (reference group: non-permanent)			
Permanent residents	−0.769** (0.337)	−0.319 (0.639)	0.027 (0.231)
Housing tenure (reference group: Owner)			
Renter	−0.676 (0.451)	−1.416* (0.807)	0.214 (0.224)
Variance Component (Neighborhood-level)	0.988	3.649	1.445
Variance Component (Individual-level)	14.075	41.009	9.633
χ 2	90.983	121.901	181.023

* p < 0.1 ** p < 0.05 *** p < 0.001; standard error in parenthesis.

**Table 8:** Modeling associations between neighborhood greens and health by MLM.

Outcome variables: Physical health			
	Model H1	Model H4	Model H7
Intercept	24.791*** (0.509)	24.783*** (0.498)	24.781*** (0.486)
Neighborhood-level variables			
Perceived park green	0.750 (0.811)		
Perceived public-square green		0.816 (1.082)	
Perceived utility green			0.027 (1.166)
Individual-level variables	Control	Control	Control
Variance Component (Neighborhood-level)	0.963	0.983	1.051
Variance Component (Individual-level)	14.077	14.076	15.376
χ2	84.615	86.218	91.004
**Outcome variables: Mental health**			
	Model H2	Model H5	Model H8
Intercept	46.142*** (0.397)	46.137*** (0.407)	46.135*** (0.430)
Neighborhood-level variables			
Perceived park green	2.471** (1.286)		
Perceived public-square green		2.614 (1.771)	
Perceived utility green			1.694 (2.095)
Individual-level variables	Control	Control	Control
Variance Component (Neighborhood-level)	2.959	3.176	3.658
Variance Component (Individual-level)	41.008	41.222	41.224
χ2	97.104	102.93	115.769
**Outcome variables: Social health**			
	Model H3	Model H6	Model H9
Intercept	18.027*** (0.194)	18.023*** (0.194)	18.023*** (0.184)
Neighborhood-level variables			
Perceived park green	2.316*** (0.405)		
Perceived public-square green		2.765*** (0.601)	
Perceived utility green			3.506*** (0.841)
Individual-level variables	Control	Control	Control
Variance Component (Neighborhood-level)	0.713	0.722	0.629
Variance Component (Individual-level)	9.634	9.636	9.633
χ2	96.215	95.278	87.844

* p < 0.1 ** p < 0.05 *** p < 0.001; standard error in parenthesis.

As shown in Table 7, male, annual household income of 100k-199k, self-employed individual and college student are significantly positively associated with better physical health, while those with age 40+ and with permanent residence status are negatively associated with physical health. In terms of mental health, male, college student, those with annual household income of 100-199k or more than 300k and self-employed are higher and enjoy better mental health, while the mental health level of people over 40 years old or those living in rental properties are lower. In terms of social health, men, self-employed, freelancer and unemployed are negatively associated with it.

According to Table 8, it is evident that different types of perceived green space play different roles in promoting residents’ health. The results show that hypotheses 2, 3, 6 and 9 cannot be rejected, and hypotheses 1, 4, 7, 5 and 8 are rejected.

The estimates from the multilevel models H1, H4 and H7 demonstrate that there is non-significant association between any type of the three green spaces and participants’ physical health when the individual variables are added. The results suggest that although there are differences in physical health among participants in 22 neighborhoods, these differences are not primarily due to differences in perceived green space. It could be that other environmental variables, or it could be that the socio-spatial differentiation of the city itself leads to clusters of people with similar self-rated physical health.

The perceived park green is positively associated with participants’ mental health (model H2), while the perceived public-square green or utility green has no significant correlation with mental health (models H5 and H8). Recreation function is one of the essential functions of park green. In other words, parks in Beijing have largely lived up to its goal in design of vegetation landscape and provision of service facilities for visitors. Their positive effect on self-rated mental health is particularly prominent, and no such an effect is detected by the other two types of green space.

All three types of green spaces are positively associated with participants' social health, (models H3, H6 and H9). Park green or public-square green provides a venue for residents to communicate and interact with each other, either on an ad hoc basis by themselves or facilitated by neighborhood organizations or other administrative units. For utility green, its positive effect on self-rated social health is likely attributable to its association with the neighborhood greening rate and building density, which may affect residents’ sense of identity and belonging for their neighborhoods.

### 5.4 Relative strengths of the associations between perceived green space and health

Since the addition of effective neighborhood-level variables reduces the neighborhood-level variance component of the MLM model, the proportional reduction in variance reflects the explanatory power of the variable. Table 9 uses the pair-wise model to compare the relative strength of the aforementioned associations between each pair of green space type and health type. When the neighborhood-level variable is perceived park green, and the outcome variables are mental health and social health, the proportional reductions in variance are 18.87% and 50.65% respectively. That is to say, the correlation between perceived park green and social health is stronger than that between perceived park green and mental health. For the same outcome variable (social health), when the neighborhood-level variables are perceived park green, perceived public-square green or perceived utility green, the proportional reductions in variance are 50.65%,50.03% and 56.39%, respectively. That indicates that perceived utility green influences social health more than the other two types of green space, whose effects have similar strength. In sum, the perceived green space has the strongest influence on residents’ social health, followed by mental health, and then physical health.

**Table 9:** Relative strengths of the relationships between neighborhood perceived green and health.

		Physical health	Mental health	Social health
Null model 1, 2 & 3	Variance Component	0.988	3.649	1.445
Model H1, H2, H3	Variance Component	–	2.959	0.713
	proportional reduction in variance		18.87%	50.65%
Model H4, H5, H6	Variance Component	–	–	0.722
	proportional reduction in variance			50.03%
Model H7, H8, H9	Variance Component	–	–	0.629
	proportional reduction in variance	–		56.39%

### 5.5 Interactions between subjective perception and objective quality in green spaces

**Fig. 6:**
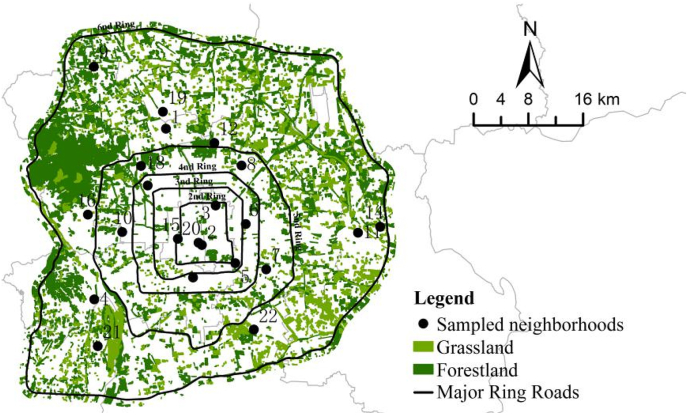
The green space coverage based on remote sensing images in Beijing.

Finally, we examine the relationship between objective green space quality and residents’ self-rated health, and the interaction between objective quality and subjective perception of green spaces. Similar to Zhang et al. (2019), this study uses the coverage of green space within a 1-km buffer based on Euclidean distance to define the objective green space for each sampled neighborhood boundary based on high-resolution remote sensing images covering Beijing (Fig. 6). Results of the extended models are reported in Table 10. There are significant positive associations between green space coverage and residents' mental health, and between green space coverage and perceived park green. The significantly positive coefficient of the product term, “Perceived park green * Green space coverage”, suggests that the objective green space coverage rate improves the residents' mental health level likely via influencing the perception of park green. A higher coverage of green space implies a lower building density of a neighborhood, more natural environment, and less crowdedness. Therefore, people tend to be more satisfied with park green, which in turn drives up the level of mental health.

**Table 10:** Extended models on associations of green space coverage and mental health.

Outcome variable: Mental health			
	Model H2a	Model H5a	Model H8a
Intercept	27.409*** (6.593)	22.193*** (6.965)	21.631*** (11.025)
Neighborhood-level variables			
Green space coverage	0.682** (0.471)	0.986** (0.581)	0.031** (0.748)
Perceived park green Perceived park green*Green space coverage	5.166** (1.853) 0.194** (0.126)		
Perceived public-square green Perceived public-square green*Green space coverage		6.511 (1.925) 0.273 (0.156)	
Perceived utility green Perceived utility green*Green space coverage			1.275 (2.914) 0.019 (0.191)
Individual-level variables	Control	Control	Control
Variance Component (Neighborhood-level)	2.163	2.475	3.593
Variance Component (Individual-level)	41.102	41.096	41.099
χ2	89.838	98.375	95.068

No significant association between green space coverage and physical health or social health are observed from our analysis, and the results of those extended models are not reported.

## 6 Discussion and conclusion

According to the joint UN-HABITAT/WHO report, all urban environments can produce “systemic, social and unfair” health inequalities, and the specific manifestations of health inequality vary from city to city and country to country (WHO, 2010). The urban development, residential setting and environmental policies in Beijing have hindered the residential mobility of its residents to some extent (Cheng et al. 2019; Shi et al. 2017; Wang et al. 2017), and poor housing affordability has confined certain population groups to neighborhoods with high exposure to environmental health risks (Shao et al. 2018; Wang & Lan, 2019; Ma et al. 2017). Without timely intervention, health inequalities in cities like Beijing will continue to grow and become detrimental to all city dwellers by disease outbreaks, social unrest, crime, and so on (WHO 2016). China has increasingly recognized the importance of health equality and environmental justice. The Healthy China initiative aims to intervene in health influencing factors and protect people's full-life-cycle health (Tan et al., 2017). As the capital city of China, Beijing is the banner bear for the initiative. However, studies on health inequality in Beijing remain scarce.

This paper is a pilot study into the spatial distribution of green spaces and their association with self-rated health in Beijing. One of the major findings is that inequality is present in all three dimensions of health at the individual and neighborhood levels. At the individual level, in addition to the apparent influence of individual socioeconomic attributes, there are significant differences in self-rated health between groups with different perceived levels of green space. The overall trend is that residents report higher levels of all three dimensions of health as their satisfaction levels with the three types of green space increase. At the neighborhood level, 6.10%, 7.23% and 12.84% of the differences in residents’ physical health, mental health and social health can be explained by the differences in perceived green space between neighborhoods. It is worth noting that social health differs the most between neighborhoods, followed by mental health and physical health.

Another significant finding is that different types of perceived green spaces play different roles in promoting residents' health. When individual socioeconomic attributes are controlled, there are non-significant correlations between any of the three types of green spaces and participants' physical health. The perceived park green is positively related to participants' mental health. All three types of perceived green space have a significantly positive association with social health. A large body of research focuses on park green and suggests that it play a vital role in influencing the health of urban residents. But our research indicates that perceived park green does not help promote self-rated physical health in Beijing residents, and its positive effect is limited to mental health and social health. By definitions, public-square green shares functions similar to park green, and is also expected to exert positive effect on residents’ health. This study only confirms its effect on social health. An in-depth investigation into this issue offers some explanation. Many residents report difficulties in access to and use of park green or public-square green so it is unlikely for them to benefit from them. According to the Beijing Gardening and Greening Bureau (BGGB 2019), the greening rate reached 48.44%, and the per-capita park green area stood at 16.3 square meters in 2018. However, both the rates are still far below the world average. The questionnaire of this study further validates this view as the most cited issue on park green and public-square green in Beijing was “small amount”, which prompted another problem of “long distance.”

The study shows that the perceived green spaces could complement each other in improving residents' social health, a major issue in public policy. Policymakers and urban planners can be more creative in improving urban green spaces while balancing with other competing measures such as high-density development and mixed land use. For example, park green usually requires a large plot of land, occupies a significant area size, and incurs high development and maintenance costs. Expanding park green space is especially challenging in high-density core areas in Beijing. Instead, investing in public-square green or utility green can be more cost effective while achieving the goal of promoting residents' interpersonal relationships and social adaptation. Those marginalized groups (e.g., the self-employed, freelance, unemployed, public housing residents, renters and those without a permanent residence status) have low social health, and could become major beneficiaries from those improvements. Overall, such a strategy can be more effective in mitigating the environmental justice in urban China. This echoes the strategy of ‘just green enough’ promoted by Curran and Hamilton (2012), and supports a cost-effective greening strategy more tailored to the have-nots.

## Funding

This research was supported by the 10.13039/501100001809National Natural Science Foundation of China (41871170, 42071215) and the Supporting Plan for Cultivating High Level Teachers in Colleges and Universities in Beijing（CIT&TCD201904075）.

## Ethical statement for SSM - population health

Hereby, we Jingxue Xu, Fahui Wang, Li Chen, Wenzhong Zhang consciously assure that for the manuscript “Perceived Urban Green and Residents’ Health in Beijing” the following is fulfilled:

1) This material is the authors' own original work, which has not been previously published elsewhere.
2) The paper is not currently being considered for publication elsewhere.
3) The paper reflects the authors' own research and analysis in a truthful and complete manner.
4) The paper properly credits the meaningful contributions of co-authors and co-researchers.
5) The results are appropriately placed in the context of prior and existing research.
6) All sources used are properly disclosed (correct citation).
7) All authors have been personally and actively involved in substantial work leading to the paper, and will take public responsibility for its content.

## Footnotes

1 Permanent resident status in Beijing, like other cities in China, is tied to the *Hukou* system and issued by the Beijing Municipal Public Security Bureau. Permanent residents are eligible for various benefits such as housing and automobile ownership, school access, health care subsidies and other social welfares, which are otherwise unavailable to non-permanent residents.

2 The Standard (MHURD, 2017) lists two other greens such as attached green (XG) and regional green (EG), which are not relevant in the context of urban neighborhoods and thus not covered in this study.

3 This strategy follows Zhang et al. (2019) and is also based on personal communications with the authors since green levels are collectively defined at the neighborhood level, even though perceived with some variability by individuals.

4 According to the classification standards of green space, the utility green does not have the functions of sight-seeing, recreation, entertainment, so the leading cause of dissatisfaction with utility green defaults to small size, and not listed in the table.
